# Cervical spondylosis with spinal cord encroachment: should preventive surgery be recommended?

**DOI:** 10.1186/1746-1340-17-8

**Published:** 2009-08-24

**Authors:** Donald R Murphy, Christopher M Coulis, Jonathan K Gerrard

**Affiliations:** 1 Rhode Island Spine Center, 600 Pawtucket Ave, Pawtucket, RI 02860-6059, USA; 2 Department of Community Health, Alpert Medical School of Brown University, Box G-A, Providence, RI 02912, USA; 3 Department of Research, New York Chiropractic College, 2360 State Rte. 89, Seneca Falls, New York 13148, USA; 4 Shoreline Spine & Pain Associates, PC, 2415 Boston Post Rd, Guilford, CT 06437, USA; 5 Clinical Sciences, University of Bridgeport, College of Chiropractic,126 Park Avenue, Bridgeport, CT 06604, USA; 6 Aquarius Chiropractic, #210-179 Davie Street Vancouver, V6Z 2Y1, USA

## Abstract

**Background:**

It has been stated that individuals who have spondylotic encroachment on the cervical spinal cord without myelopathy are at increased risk of spinal cord injury if they experience minor trauma. Preventive decompression surgery has been recommended for these individuals. The purpose of this paper is to provide the non-surgical spine specialist with information upon which to base advice to patients. The evidence behind claims of increased risk is investigated as well as the evidence regarding the risk of decompression surgery.

**Methods:**

A literature search was conducted on the risk of spinal cord injury in individuals with asymptomatic cord encroachment and the risk and benefit of preventive decompression surgery.

**Results:**

Three studies on the risk of spinal cord injury in this population met the inclusion criteria. All reported increased risk. However, none were prospective cohort studies or case-control studies, so the designs did not allow firm conclusions to be drawn. A number of studies and reviews of the risks and benefits of decompression surgery in patients with cervical myelopathy were found, but no studies were found that addressed surgery in asymptomatic individuals thought to be at risk. The complications of decompression surgery range from transient hoarseness to spinal cord injury, with rates ranging from 0.3% to 60%.

**Conclusion:**

There is insufficient evidence that individuals with spondylotic spinal cord encroachment are at increased risk of spinal cord injury from minor trauma. Prospective cohort or case-control studies are needed to assess this risk. There is no evidence that prophylactic decompression surgery is helpful in this patient population. Decompression surgery appears to be helpful in patients with cervical myelopathy, but the significant risks may outweigh the unknown benefit in asymptomatic individuals. Thus, broad recommendations for decompression surgery in suspected at-risk individuals cannot be made. Recommendations to individual patients must consider possible unique circumstances.

## Background

Degenerative changes in the cervical spine are part of the normal aging process and are nearly ubiquitous in older people [1]. They are generally asymptomatic [2,3]. Spondylosis, with the development of osteophytes, occurs as part of the degenerative process. This can lead to the development of clinical symptoms in some individuals if the osteophytes impinge on neural structures such as the nerve root or spinal cord. If this encroachment occurs in the lateral recess or lateral canal it can lead to radiculopathy. If it occurs in the central canal it can cause myelopathy. However, encroachment in either of these regions can also be asymptomatic with regard to myelopathy [1,4]. For example, Matsumoto, et al [1] assessed 497 asymptomatic subjects and found posterior disc protrusion with compression of the spinal cord in 7.6%. While this figure was presented in the abstract of the paper, no details were provided as to how this compression was measured. However, the figure was similar to that of Teresi, et al [5] who found cord compression on MRI in 7 of 100 asymptomatic subjects. Cord compression without myelopathy has also been found on CT myelography [6].

Cervical spondylotic myelopathy (CSM) is the most common cause of spinal cord dysfunction in older individuals and usually develops insidiously [7]. However, it has been reported to develop after trauma [8-15]. Some authors have suggested that individuals who have asymptomatic spondylotic encroachment on the cervical spinal cord are at increased risk of acute myelopathy if they experience minor trauma such as a fall or motor vehicle collision [16,17]. This has led some surgeons to recommend decompression surgery for the purpose of preventing this trauma-induced myelopathy in presumed susceptible individuals [18,19]. For example, Epstein [18] stated "Patients under 65 years of age, if mildly symptomatic or at risk for quadriplegia with even mild trauma, may warrant early decompression". However, he did not provide evidence-based recommendations as to how to determine risk of quadriplegia or the level of risk that would warrant surgery in the absence of frank myelopathy.

The authors, all non-surgical spine specialists, have had patients consult them for second opinion after being recommended this type of surgery. Each of these patients was asymptomatic with regard to cervical myelopathy (though they had neck pain), but cervical MRI had revealed cervical spondylosis which encroached on, and compressed, the spinal cord. It was reported in each of these cases that the surgeon making the recommendation did so based on the view that the spinal cord encroachment placed the patient at risk of spinal cord injury if he or she were to experience even relatively minor trauma. These patients expressed a desire for a non-surgical opinion as to whether such surgery is truly advisable. This is apparently a frequent enough occurrence in the experience of other spine specialists to have warranted a "Curve/Countercurve" piece in a recent issue of Spine Line, a publication of the North American Spine Society [19].

Evidence-based medicine calls for the clinician to provide counseling and treatment that is based on the best available evidence, combined with clinical experience and patient preference [20-22]. The purpose of this review is to investigate whether the scientific literature can be used to inform the surgical and non-surgical spine specialist regarding how to advise patients who have spondylotic encroachment on the cervical spinal cord in the absence of frank myelopathy.

## Methods

The following databases were searched up to May 31, 2008: Medline, Cinahl, Embase and MANTIS. Searches of the authors' own libraries were also conducted. Finally, citation searches of relevant articles and texts were conducted manually. The search terms used for the database searches can be found in table 1.

**Table 1:** Search terms

Search Terms for Risk of Spinal Cord Injury	Search Terms for Surgery
"cervical spondylosis" AND whiplash	"cervical myelopathy" AND surgery AND risk
"cervical spondylosis" AND trauma	"cervical laminectomy" AND surgery AND risk
"cervical spondylosis" AND risk AND whiplash	"cervical myelopathy" AND surgery AND complications
"cervical myelopathy" AND whiplash	"cervical myelopathy" AND surgery
"cervical myelopathy" AND trauma	"cervical laminectomy"
"cervical spondylosis" AND "cervical myelopathy" AND whiplash	"cervical decompression" AND surgery

The search yielded 1881 citations. Relevant papers were retrieved and reviewed by two independent reviewers. Studies that were deemed relevant were those that investigated the risk of spinal cord injury from minor trauma in patients with pre-existing spondylotic central canal encroachment and those that reported on outcomes and complications to cervical decompression surgery, with or without fusion. Case reports and small case series were excluded. Also excluded were studies reporting risk of spinal cord injury resulting from major trauma and studies involving individuals who had narrowing of the central canal from sources other than degenerative changes. In cases in which systematic reviews of the literature were found, the individual studies included in the reviews were not reviewed separately, unless this was necessary to clarify information that was not readily apparent from the systematic review.

## Results

### Risk of Spinal Cord Injury from Minor Trauma

Five studies [9-11,13,23] were excluded because they assessed younger individuals in whom degenerative spondylotic change would not be expected. One study that excluded subjects with cervical spondylosis was also excluded from the present study [24]. Three studies were excluded because all of the subjects [25,26] or more than half [12] had major trauma (fracture and/or dislocation). One study was excluded because it looked at rate of recovery and not incidence or risk [27]. Two studies met the inclusion criteria [14,15].

Regenbogen, et al [14] retrospectively reviewed the medical records of 88 patients over age 40 with spinal cord injury resulting from trauma and compared them with a group of 35 young adults (16–36 years) with spinal cord injury. Of the 88 older patients, 25 had no bony or ligamentous injury and another 17 had "subtle" signs of bony or ligamentous injury. In contrast, only one of the 35 younger patients had developed spinal cord injury without severe bony or ligamentous injury. All 25 patients with no bony injury were evaluated with radiographs and 16 with pantopaque myelography. All patients imaged with myelography had signs of "moderate to severe" spondylosis. Katoh, et al [15] reported on 27 patients with ossification of the posterior longitudinal ligament who sustained minor trauma ("such as tumbling, slipping or jumping from small steps") to the cervical spine. Thirteen of these patients developed new myelopathy, 7 experienced deterioration of pre-existing myelopathy and 7 experienced no neurologic sequelae. Eighteen of the 19 patients with a narrow central canal (<10 mm) developed neurologic deterioration, whereas this occurred in only two of the eight patients with a wider canal (10 mm or greater).

### Benefits and Risks of Surgery in the Cervical Spine in Asymptomatic Spinal Cord Encroachment

The search did not reveal any studies on the outcome of surgery in asymptomatic or presumed "at risk" subjects. It did reveal a number of review papers [28-34] that included most of the studies found in the search. The most common surgical procedures used in this patient population are discectomy, laminectomy with or without foraminotomy or fusion, circumferential decompression with fusion, laminoplasty and corpectomy. Each has its own indications and contraindications as well as complications. These are provided in Table 2. Potential complications to these surgical procedures include injury to the spinal cord, nerve roots, sympathetic ganglia, recurrent laryngeal nerve, or vertebral artery, CSF leakage, infection and pseudoarthrosis (Table 2).

**Table 2:** Surgical procedures for cervical spondylotic myelopathy

Procedure	Indications	Contraindications	Complications
Discectomy [28]	Radiculopathy; Myelopathy; Myelo-radiculopathy; Traumatic instability involving single or multiple levels	Increased age Posterior cord/canal pathology	Recurrent laryngeal nerve injury -0.07 to 24.2%; Dysphagia – 12.3%; Hoarseness – 4.9%; unilateral vocal cord impairment -1.4%; Neurological complications – 0.3%; Pseudoarthrosis -6.9%*

Laminectomy with fusion [29]	Multi-level (> 3 segments), myelopathy	Cervical kyphosis	Cervical kyphosis -21%; Hypermobility; Spinal cord injury -3%; Nerve root injury -15%; Penetration of vertebral artery -5.8–6.7%

Circumferential decompression with fusion [30]	Bicolumnar failure; Flexion-compression injury; Burst fracture; Poor bone quality; More stable construct; decreases use of halo; improved graft fusion	Increased age	Vertebral fracture and graft extrusion; Fixed plate failure warranting revision surgery – 13%; Posterior wound failure – 3%

Laminoplasty [32,42]	Multilevel spondylosis and OPLL	Cervical kyphosis Poor results with 1–2 level decompression	Loss of lordosis – 22–53%; Kyphosis – 2–4%; Loss of ROM; decrease 17–50% and >70% with fusion; Infection; Fracture of the "hinged" side can lead to spinal cord injury; Axial neck pain -6–60%; Nerve root palsy 1–3 days post-op, predominantly motor loss of C5 – 11% (6.8% at 2 year follow-up)

Corpectomy [31]	Multi-level disease; Extends behind posterior vertebral body; Severe osteophytosis; VB deformity	Increased age Posterior canal/cord pathology	Recurrent laryngeal nerve injury; CSF leakage; Sympathetic ganglion injury; Perforation of esophagus – 0.25%; Dysphagia – 45%; Veterbal artery injury – 0.3%; Bone graft complication; pseudoarthrosis – 7% with single level fusion and 30% with 3 level fusion

*rate increases with each segmental level added

## Discussion

The role of preventive surgery in patients with asymptomatic cervical spinal cord encroachment has been a point of controversy amongst surgeons. Riew, in a point-counterpoint piece, [19] argued that the risk of myelopathy in patients with asymptomatic encroachment on the cervical spine is not worth the risk of surgery. Combining data from the Paralyzed Veterans of America, National Library of Medicine, and the US Census, he estimated the "worst case scenario" risk of myelopathy in this patient population to be 1:2100. He argued that even if the risk of serious complication from surgical decompression was 1:1000, this would be twice the risk of myelopathy after trauma [19]. As has been pointed out in the present paper, however, the studies Riew cited on which he based the assumption of risk were of inadequate design to assess true risk [25,26]. However, this point only strengthens his recommendation against surgery in this population. Others [18] have argued that because of the potentially catastrophic nature of spinal cord injury after trauma, decompression surgery is appropriate in this patient population. The purpose of this study is to assess the evidence regarding this risk and attempt to compare what is known about this risk with what is known about the risk of surgery. It is hoped that all spine clinicians can take an evidence-based approach to counseling patients with this condition.

All studies that related to the risk of spinal cord injury in patients with asymptomatic encroachment located in the search were case reports, case series or retrospective cross-sectional studies. None were case-control or prospective cohort studies. Thus, while it can be said that there may be an association between the presence of asymptomatic cord encroachment and spinal cord injury after trauma, no firm conclusions can be drawn about causation. Case-control or prospective cohort studies would be necessary to make this determination [35]. Also, in the majority of cases the size of the central canal was measured with radiographs. Recent evidence indicates poor correlation between radiographically-determined central canal size and that determined by MRI [36]. Because the studies were of inadequate design to assess risk and used inadequate measurement methods, the present authors did not feel that it was of benefit to undergo a formal critical appraisal of the studies.

Bednarik, et al [37,38] have studied risk factors for the development of CSM in individuals with asymptomatic spondylotic cord compression using a prospective cohort design. In their initial study of 66 subjects with this condition who were followed for 2–8 years [37], they found that 13 subjects (19.7%) developed symptomatic CSM. The only risk factors for the progression to CSM in this cohort were symptomatic radiculopathy at baseline, electromyographic (EMG) evidence of anterior horn lesion at baseline and abnormal somatosensory evoked potentials (SSEP) at baseline. In a more recent publication with a larger sample size (n = 199) and longer follow period (2–12 years, median 44 months) [38] they found that 45 subjects (22.6%) developed symptomatic CSM. Baseline symptomatic radiculopathy, EMG evidence of anterior horn cell lesion and abnormal SSEP were found to be risk factors for the development of CSM during the follow up period. There was a tendency toward increased risk in males *vs *females and in those with abnormal motor evoked potentials, but these did not reach statistical significance (*p *= 0.072 and *p *= 0.112, respectively). Factors in their model that were not found to increase risk of the development of CSM were age, type of compression (spondylosis, disc herniation or the combination of both), number of stenotic levels, decreased cross sectional area of the spinal canal, decreased Pavlov ratio and hyperintense signal within the spinal cord on T2-weighted MRI image. They did not include exposure to trauma in their analysis, however, when re-analyzing the data they found relatively few exposures to trauma and that these had no impact on development of CSM (Bednarik J, personal communication 26^th ^June 2008).

In all the surgical studies found in the search, the subjects had symptomatic myelopathy. No outcome studies were found that included asymptomatic subjects thought to be at risk. Thus, the role surgery plays in preventing spinal cord injury in asymptomatic subjects thought to be at risk is not known. It is also not known whether the complication rate of decompression surgery in patients with asymptomatic cord encroachment would be the same as in those with myelopathy. However, as the reported postsurgical complications generally relate to the surgery itself and not to the myelopathy (see Table 1), it is not likely that the complication rate would be substantially different in asymptomatic individuals as compared to symptomatic individuals.

Based on this review of the literature, it remains to be determined whether an individual with cervical spinal cord encroachment, without signs or symptoms of myelopathy, is at increased risk of spinal cord injury after trauma. It also remains to be determined what the magnitude is of any increased risk. This determination would require population-based case-control or, preferably, prospective cohort studies. With these designs, bias can be minimized and statistical conclusions can be drawn regarding risk [35]. Until such studies have been performed, it cannot be stated with certainty that individuals with the findings discussed here are at increased risk of trauma-induced myelopathy.

Because of this, there is currently no substantial evidence upon which to base a recommendation for prophylactic decompression surgery in this patient population. However, evidence-based medicine calls for recommendations to be individually directed and to take into account scientific evidence combined with clinical experience and patient preference [20-22]. There may be individual variations in a particular case, such as severe canal encroachment, low signal change within the spinal cord on T1 weight images with high signal on the T2 weighted images (which has been found to correlate with poor surgical outcome) [39], ossification of the posterior longitudinal ligament or persistent engagement in high-risk activities, which may influence one's recommendation. Also it may be advisable for the non-surgical spine specialist to counsel patients who have asymptomatic cord encroachment to avoid high-risk activities, particularly those that could involve high-acceleration extension injury. Given the fact that post-traumatic myelopathy has been reported to be associated with falls in the elderly [40], it would be reasonable for elderly patients with this finding to be provided prevention strategies, including exercises for improved balance, in order to lessen the likelihood of falling [41].

## Conclusion

Asymptomatic cervical spondylotic spinal cord encroachment is fairly common. It has been said that individuals with this finding are at increased risk of severe myelopathy if they experience minor trauma. In some cases, prophylactic decompression surgery has been recommended. However, there is no good evidence that these individuals are at increased risk and, given the potentially serious complications of surgery, the evidence does not allow for firm and broad recommendations to be made regarding prophylactic surgery. Population-based case-control or prospective cohort studies are needed to determine whether the magnitude of any risk in this patient population justifies surgical intervention.

## Competing interests

The authors declare that they have no competing interests.

## Authors' contributions

DRM conceived of the research idea, supervised the literature search and data extraction process and was the principle writer of the manuscript. CMC and JKG conducted the literature searches and were involved in data extraction. All authors reviewed and made editorial changes in the manuscript. All authors read and approved the final manuscript.
